# Molecular Detection of Avian Pathogenic *Escherichia coli* (APEC) for the First Time in Layer Farms in Bangladesh and Their Antibiotic Resistance Patterns

**DOI:** 10.3390/microorganisms8071021

**Published:** 2020-07-09

**Authors:** Samina Ievy, Md. Saiful Islam, Md. Abdus Sobur, Mithun Talukder, Md. Bahanur Rahman, Mohammad Ferdousur Rahman Khan, Md. Tanvir Rahman

**Affiliations:** Department of Microbiology and Hygiene, Faculty of Veterinary Science, Bangladesh Agricultural University, Mymensingh 2202, Bangladesh; v.samina@gmail.com (S.I.); dvm41257@bau.edu.bd (M.S.I.); soburvetbau@gmail.com (M.A.S.); saddomithun@gmail.com (M.T.); bahanurr@bau.edu.bd (M.B.R.); mfrkhan@bau.edu.bd (M.F.R.K.)

**Keywords:** avian pathogenic *E. coli*, virulence genes, multidrug-resistant, layer farm, air, public health

## Abstract

Avian pathogenic *Escherichia coli* (APEC) causes significant economic losses in poultry industries. Here, we determined for the first time in Bangladesh, the prevalence of APEC-associated virulence genes in *E. coli* isolated from layer farms and their antibiotic resistance patterns. A total of 99 samples comprising internal organs, feces, and air were collected from 32 layer farms. Isolation was performed by culturing samples on eosin–methylene blue agar plates, while the molecular detection of APEC was performed by PCR, and antibiograms were performed by disk diffusion. Among the samples, 36 were positive for the APEC-associated virulence genes *fimC*, *iucD,* and *papC*. Out of 36 isolates, 7, 18, and 11 were positive, respectively, for three virulence genes (*papC*, *fimC*, and *iucD*), two virulence genes, and a single virulence gene. Although the detection of virulence genes was significantly higher in the internal organs, the air and feces were also positive. The antibiograms revealed that all the isolates (100%) were resistant to ampicillin and tetracycline; 97.2%, to chloramphenicol and erythromycin; 55.5%, to enrofloxacin; 50.0%, to norfloxacin and ciprofloxacin; 19.4%, to streptomycin; 11.1%, to colistin; and 8.33%, to gentamicin. Interestingly, all the isolates were multidrug-resistant (MDR). Spearman’s rank correlation coefficient analysis revealed the strongest significant correlation between norfloxacin and ciprofloxacin resistance. This is the first study in Bangladesh describing the molecular detection of APEC in layer farms. Isolated APEC can now be used for detailed genetic characterization and assessing the impact on public health.

## 1. Introduction

Poultry farming is a well-developed and profitable agri-business in Bangladesh. It is estimated that the livestock and poultry sector contributes around 1.47% of the total GDP of the country [1]. Poultry products including eggs and meat chiefly fulfil the daily protein requirements of Bangladesh’s population. However, from time to time, the advancement of poultry production is seriously hampered by various infectious diseases [2]. Among the infectious agents, avian pathogenic *Escherichia coli* (APEC) is thought to be prevalent in Bangladesh. Although *E. coli* is part of the normal bacterial microbiota of the intestinal tract, other mucosal surfaces of hosts, and the poultry farm environment, few of these strains are endowed with specific virulence factors that define the APEC phenotype [3]. Most APEC strains are phylogenetically associated with extra-intestinal location [4,5]. Principally, they are associated with respiratory tract and systemic infections [6]. In addition, APEC respiratory infections are secondary to other respiratory tract infections, including infectious bronchitis virus (avian coronavirus), Newcastle disease virus, and *Mycoplasma gallisepticum* [7].

Avian colibacillosis, caused by APEC, is a complex syndrome that has an ominous impact on the poultry sector worldwide [8]. Avian colibacillosis is characterized by multiple organ lesions such as air sacculitis, pericarditis, peritonitis, salpingitis, synovitis, osteomyelitis, yolk sac infections, etc. [9,10]. Thus, APEC is a major cause of extensive economic loss in the poultry industry due to high morbidity and mortality [4]. Both the broiler and layer farms are affected by APEC. Several of the characteristic virulence associated genes (VAGs) of APEC are *iss*, *kps*, *cvi*, *tss*, *papC*, *fimC*, *iucD*, etc. [6,11]. Virulence factors (invasins, adhesins, iron acquisition systems, toxins, and protectins) coded by multifarious VAGs facilitate the infection-causing abilities of *E. coli* strains [8]. These virulence factors may become useful for pathogenic strains of *E. coli* by enabling invasion, colonization, and adherence and protecting *E. coli* from host defenses [12,13,14]. Among them, *papC* (pyelonefritis associated to pili C) is associated with the production of adhesion-related factors to enable the adhesion of *E. coli* and is responsible for occurring infections [8,15], *fimC* (Type 1 fimbriae C) is connected with the adhesion and colonization of *E. coli* on epithelial cells [16], and *iucD* (iron-uptake systems of *E. coli* D) demonstrates the difference between APEC and non-APEC isolates in terms of the iron acquisition system [17]. The virulence of any particular isolate of *E. coli* correlates with the number and combination of these virulence-associated genes [18]. These virulence factors may be found as single genes or as associations of different gene combinations in both healthy and clinical isolates [19].

Along with APEC, uropathogenic *E. coli* (UPEC) and neonatal meningitis *E. coli* (NMEC) are also considered as extra-intestinal pathogenic *E. coli* (ExPEC). In humans, ExPEC may cause urinary tract infections (UTIs), neonatal meningitis, and septicemia [20,21]. Phylogenic similarities among APEC, UPEC, and NMEC strains—transmissible plasmids, virulence genes, and other genetic characteristics—indicate that APEC are zoonotic in nature [21,22]. In addition, APEC are present in the intestines and meat of healthy poultry, possessing genetic similarities with human ExPEC, indicating a possible transmission of animal APEC to humans [22]. Some experimental studies have expounded the transmission pattern between avian ExPEC and human ExPEC with a negative impact on public health [23].

Antimicrobial resistance (AMR) is an ever-increasing public health crisis. The G20 partners have recognized AMR as a major “growing threat to public health and economic growth”. It causes an estimated 700,000 deaths each year across the world [24]. Drug-resistant APEC strains can contaminate the food supply from farm to fork through eggs, meat, and other contaminants and thus pose a severe threat to the consumer’s health [25]. The indiscriminate use of antibiotics in poultry production may have contributed to drug resistance in APEC. From the poultry farm, drug-resistant strains are deposited into soil, wastewater, air, and the environment [26].

Studies from many countries have detected drug resistance determinants in APEC [27,28]. In Bangladesh, previous studies have identified antibiotic-resistant *E. coli* from poultry [29,30,31,32]. These studies, however, did not focus on APEC and the associated virulence genes. Therefore, the present study was designed to determine the prevalence of APEC and the associated virulence genes in *E. coli* in layer farms in Bangladesh, as well as their antibiotic resistance profile.

## 2. Materials and Methods

### 2.1. Ethics Statement

The experimental procedures and protocols used in this study were approved by the Animal Welfare and Experimentation Ethics Committee of Bangladesh Agricultural University (approval number AWEEC/BAU/2019(28)). 

### 2.2. Sample Collection and Processing

Samplings were done in January–November 2019 from 32 layer farms located in Mymensingh district, Bangladesh. A total of 99 samples were collected aseptically, comprising seven different types of samples, including air from the insides of poultry shades (*n* = 31), feces from sick birds (*n* = 32), and the intestinal organs (trachea, intestine, liver, lung, and egg yolk material; *n* = 36) of dead birds. Air sampling was done using the settle plate method as previously described by other [33] with some modifications. In brief, instead of nutrient agar, here, eosin methylene blue (EMB) agar plates were exposed at 1 m above the ground to different corners of the poultry shades for 10 min. Freshly dropped fecal samples were collected using sterile cotton buds from sick isolated groups of birds. Internal organs were collected during post-mortem examinations. All the collected samples were given unique tag numbers and transported to the laboratory maintaining the cold chain. Immediately after arrival at the laboratory, fecal samples (1 g) were seeded into test tubes containing 5 mL of nutrient broth. Internal organs were initially cut into small pieces and then transferred into test tubes containing 5 mL of nutrient broth. EMB agar plates and test tubes were then incubated aerobically at 37 °C overnight.

### 2.3. Isolation and Identification of E. coli

The isolation and identification of *E. coli* was based on culture on EMB agar plates. For this purpose, the overnight-grown broth cultures were streaked on EMB agar plates and incubated aerobically at 37 °C overnight. Single metallic sheen colonies on the EMB agar plates were considered as indicative of *E. coli*. These colonies were then subjected to morphological study by Gram staining, basic sugar fermentation tests, methyl red tests, Voges–Proskauer tests, and indole tests [34]. The final confirmation of the isolation of *E. coli* was performed by polymerase chain reactions (PCRs) targeting the *E. coli* 16S rRNA gene [35].

For PCR, genomic DNA was extracted from *E. coli* pure cultures by the boiling method [36]. In brief, a pure colony was put into an Eppendorf tube containing 100 µL of deionized water and gently vortexed, followed by boiling and cooling for 10 min during each step. Finally, genomic DNA was collected after centrifugation for 10 min and stored at −20 °C for further use.

### 2.4. Molecular Detection of APEC-Associated Virulence Genes

Several genes are known to be associated specifically with APEC. In this study, we selected the *fimC*, *iucD*, and *papC* genes as the molecular markers for the detection of APEC. These are the commonly APEC-associated virulence genes detected in the majority of the studies focused on APEC (8, 22, 60, 61, 62, 74). Moreover, as mentioned earlier, *iucD* differentiates APEC from non-APEC (17). Once confirmed, isolated *E. coli* were screened by PCR for detecting the APEC-associated virulence genes *fimC*, *iucD,* and *papC* [12]. The primers used for the detection of APEC are listed in Table 1.

PCR tests were done in a final 25 µL reaction with 12.5 µL of master mix (2X) (Promega, Madison, WI, USA), 4 µL of genomic DNA (50 ng/µL), 1 µL of each primer, and 6.5 µL of nuclease-free water. After completion, the amplified PCR products were analyzed by electrophoresis in 1.5% agarose. Amplicons were stained by ethidium bromide and visualized under an ultraviolet trans-illuminator (Biometra, Göttingen, Germany). A 100 bp DNA ladder (Promega, Madison, WI, USA) was used to check the size of the PCR amplicons.

### 2.5. Antimicrobial Susceptibility Test

Isolated *E. coli* positive for APEC-associated virulence genes were used for disk diffusion tests as reported [37]. Ten commonly used antibiotics of different classes were employed: penicillins (ampicillin—2 µg disk), amphenicols (chloramphenicol—10 µg), fluoroquinolones (ciprofloxacin—5 µg; enrofloxacin—10 µg; and norfloxacin—10 µg), polypeptides (colistin—10 µg), macrolides (erythromycin—15 µg), aminoglycosides (gentamicin—10 µg; and streptomycin—10 µg), and tetracycline (tetracycline—30 µg). Antimicrobial susceptibility tests (ASTs) were performed on Mueller–Hinton agar plates (Himedia, India) with a concentration of freshly grown bacteria equal to 0.5 McFarland units. The results were recorded as sensitive, intermediate, or resistant as per the standards of the Clinical and Laboratory Standards Institute [38]. Multidrug-resistant (MDR) isolates were categorized according to Sweeney et al. [39].

### 2.6. Statistical Analysis

The results were inserted into an Excel 2013 spreadsheet (Microsoft Office 2013, Microsoft, Los Angeles, CA, USA) and analyzed using the SPSS software (IBM SPSS version 25.0, IBM, Chicago, IL, USA). Descriptive analysis was conducted to calculate prevalence. A chi-square test for relatedness was done to determine the possible relationships of the sample type with the prevalence of *E. coli* and APEC-associated virulence genes. The chi-square test for goodness-of-fit was applied to observe if any differences existed among the frequencies of the three APEC-associated virulence genes. A *p*-value less than 0.05 (*p*-value < 0.05) was considered as statistically significant.

In addition, a Spearman rank correlation with a Bonferroni correction (*α*/8) was performed to determine the possible pairwise correlation among various antimicrobial resistance patterns using a piece of statistical software named STATA (STATA version 16.0) as previously described by Varga et al. [40]. A *p*-value less than or equal to 0.00625 (*p* ≤ 0.00625; *α*/8) indicated the test result as statistically significant.

## 3. Results

### 3.1. Prevalence of E. coli Isolates

Among 99 samples, 82 (82.83%) were positive for *E. coli* according to the PCR targeting of the *E. coli* 16S rRNA gene. The highest prevalence was found in feces (100%), and the lowest, in air samples (67.74%). The overall prevalence of *E. coli* in various samples is presented in Table 2. Statistical analysis revealed that feces carried a significantly higher percentage of *E. coli* than the other samples investigated (chi-square test, 95% CI, *p* = 0.003).

### 3.2. APEC-Associated Virulence Genes

Out of 82 *E. coli*, 36 (36.36%) were positive for APEC-associated virulence genes (Table 3). A significantly higher prevalence of APEC-associated virulence genes was observed in the internal organs than in air (16.13%) and feces (21.87%) (chi-square test, 95% CI, *p* < 0.001).

Among the 36 *E. coli* isolates carrying APEC-associated virulence genes, seven were positive for three virulence genes (*fimC*, *iucD,* and *papC*); 18 were positive for two virulence genes (in different combinations), and 11 were carrying a single virulence gene (Table 3). The most prevalent combination was *fimC*/*iucD* (in 11 isolates); however, *fimC*/*papC* was in three isolates. Statistical analysis indicated that *fimC* (97.22%) was significantly more prevalent than *iucD* (58.33%) and *papC* (33.33%) in 36 *E. coli* (chi-square test, 95% CI, *p* = 0.003).

### 3.3. Antibiogram Profile of E. coli Isolates Carrying APEC-Associated Virulence Genes

**Figure 1:**
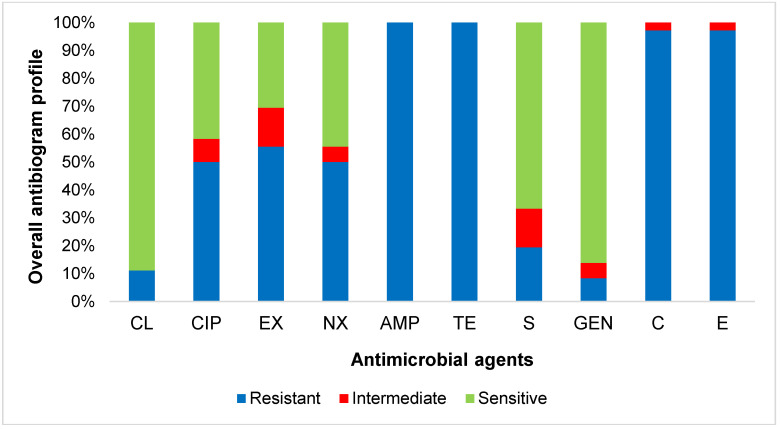
Overall antibiogram profile of the isolated APEC. CL, Colistin; CIP, Ciprofloxacin; EX, Enrofloxacin; NX, Norfloxacin; AMP, Ampicillin; TE, Tetracycline; S, Streptomycin; GEN, Gentamicin; C, Chloramphenicol; E, Erythromycin.

**Figure 2:**
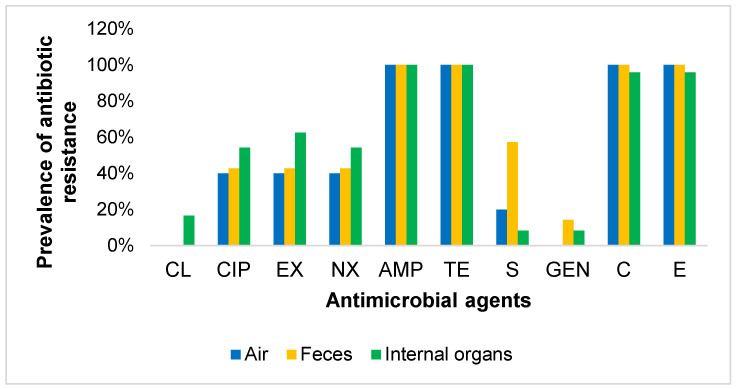
Sample wise antibiotic resistance profile of the isolated APEC. CL, Colistin; CIP, Ciprofloxacin; EX, Enrofloxacin; NX, Norfloxacin; AMP, Ampicillin; TE, Tetracycline; S, Streptomycin; GEN, Gentamicin; C, Chloramphenicol; E, Erythromycin.

An antibiogram study showed that all 36 *E. coli* isolates carrying APEC-associated virulence genes were resistant to ampicillin and tetracycline (100%), followed by resistance to chloramphenicol and erythromycin (97.2%), to enrofloxacin (55.5%), to norfloxacin and ciprofloxacin (50.0%), to streptomycin (19.4%), to colistin (11.1%), and to gentamicin (8.3%) (Figure 1). Detailed results of the AST and sample-wise antibiotic resistance profile of APEC are presented in Table 4 and Figure 2, respectively. All the APEC isolates were MDR in nature (Table 5).

A total of 16 antibiotic resistance patterns were observed among the APEC isolates. Among the antibiotypes, resistance pattern no. 1 (AMP, TE, C, E) was the most prevalent (30.5%), followed by pattern no. 9 (CIP, EX, NX, AMP, TE, C, E) in 16.7% of the isolates and pattern no. 12 (CIP, EX, NX, AMP, TE, S, C, E) in 11.1% of the isolates. One isolate (Io-Lu2) showed resistance to nine antibiotics (seven classes of antimicrobials) of the ten tested.

### 3.4. Pairwise Correlation between Resistance to Antimicrobials

Statistical analysis revealed that norfloxacin and ciprofloxacin resistance showed the strongest significant correlation (Spearman’s rank correlation coefficient, ρ = 0.8315 with *p*-value = 0.0000), followed by a significant correlation between enrofloxacin and ciprofloxacin resistance (ρ = 0.6625 with *p*-value = 0.0000) and norfloxacin and enrofloxacin resistance (ρ = 0.6203 with *p*-value = 0.0001). The pairwise correlations among the resistance to antimicrobials are represented in Table 6.

## 4. Discussion

APEC-associated avian colibacillosis has a significant impact on the poultry industry. APEC are also important from the public health point of view [23]. Previous studies have indicated avian colibacillosis as a prominent disease of commercial chickens [17,21]. Globally, poultry industries are being confronted by enormous economic losses due to the dramatic impact of the disease [41]. To control colibacillosis, multiple antimicrobials have been used indiscriminately, especially in middle- and low-income countries including Bangladesh, contributing to the development and spread of AMR. The subsequent selection of MDR strains has generated serious challenges in terms of public health. Sustainable development goals (SDGs) are affected by AMR, especially in targeting hunger, poverty, malnutrition, health, and economic growth [24]. Thus, investigations of APEC strains with regard to virulence genes and AMR profiles may help to curtail their hazardous effects.

In this study, the overall prevalence of *E. coli* was found to be 82.8%. This is in agreement with Hadiujjaman et al. [42] and Islam et al. [43], who reported the *E. coli* prevalence in layers to be 80% and 85% in Bangladesh. Al Azad et al. [44] reported an isolation rate of *E. coli* of 100% in poultry, which is rather higher than ours. However, the observed variation in the prevalence of *E. coli* in poultry farms may be linked with differences in isolation methods, geographic locations, hygienic practices, sanitation, and other management practices in farms. A significantly higher occurrence of *E. coli* in the fecal samples (100%) than in the air from inside poultry shades (67.74%) and internal organs of layer birds (80.56%) is justified, as *E. coli* is part of the normal microbiota of the digestive tract. Several studies from home and abroad have also detected *E. coli* in internal organs [17,45,46,47], the air from inside poultry shades [48], and feces [49].

The current study is chiefly concerned with APEC, which is determined by virulence gene detection. Previously similar investigations have been undertaken across the globe [40,50,51]. According to Chui et al. [52], virulence genes can be used as molecular markers for the detection of specific groups of pathogens. In this study, APEC strains were identified through the detection of the virulence genes *fimC*, *iucD*, and *papC* [12,53]. The overall prevalence of APEC was 36.4%, with a significantly higher occurrence in internal organs (66.7%) than in feces (21.9%) and air (16.1%) samples. A high prevalence of APEC in internal organs such as the trachea, lungs liver, intestine, and egg yolk is not unusual since the current study collected these organs from dead birds of sick groups. In addition, APEC—as opportunistic pathogens—usually cause secondary infections in internal organs during the occurrence of infectious bronchitis, Newcastle disease, mycoplasmosis, and others [7]. Previously, several studies had detected APEC in the egg yolk [54], trachea [55], lung epithelia [56], liver [17], and intestine [57]. Although APEC are mostly ExPEC, the presence of APEC in the intestine demonstrates the intestinal colonization of *E. coli*. According to Dho-Moulin [3], APEC are found in the intestinal microbiota of healthy birds showing no disease symptoms. In fact, intestinal microbiota can act as reservoirs for APEC [57]. Therefore, the fecal presence of APEC is not unusual. We also detected APEC in the air from the inside of poultry shades, which may be associated with the fecal contamination of air [48]. Previously, Obeng et al. [58] and Stromberg et al. [49] recorded the prevalence of APEC in fecal samples from layers and broilers as 10% and 13%. Kogovšek et al. [59] revealed an association of APEC virulence genes with air samples. This evidence shows that the APEC strains harbored by commercial chickens and their environment can be transmitted from bird to bird and farm to farm through a variety of ways, including air, feces and utensils used in farms. Moreover, APEC strains are zoonotic in nature, and their presence in air and feces within a farm with which humans are associated is a health issue; hence, they pose a great risk for human health [23].

The present study showed that the APEC-associated gene *fimC* was significantly more prevalent (97.2%) than *iucD* (58.3%) and *papC* (33.3%). A similar order of prevalence of *fimC* (92.7%) > *iucD* (78.79%) > *papC* (22.73%) and *fimC* (96.97%) > *iucD* (82.7%) > *papC* (30%) was reported by Janßen et al. [12] and Paixao et al. [8], respectively. In addition, the higher prevalence of *fimC* compared to that of other virulence genes has been detected in different countries—e.g., 93.6% *fimC*, 70.8% *iucD*, and 6.5% *papC* in China [60]; 95% *fimC*, 71.65% *iucD*, and 36.65% *papC* in Japan [61]; 94.87% *fimC* and 8.69% for both *iucD* and *papC* in Italy [62]; and 92% *fimC*, 72% *iucD*, and 48% *papC* in Pakistan [63]. However, both *fimC* and *papC* are responsible for the adhesion of *E. coli* to cells [8,15]. The *fimC* gene plays a greater role in adhesion. The presence of *iucD* promotes the survival of APEC, as the gene is associated with iron, which is essential for *E.coli* survival [64]. Two or more APEC-associated virulence genes were detected at 30.5% among the *E. coli* isolated in this study (25/82), while 10% of the isolates had the same pattern as that identified by Obeng et al. [58]. The detection of virulence genes singly or in combination from clinical samples shows that *fimC*, *iucD,* and *papC* are pivotal virulence genes of APEC strains.

Antimicrobials are widely used as a primary measure to control APEC infections and reduce economic losses globally, particularly in the developing world, including Bangladesh. The antibiotics used in the farm themselves act as a selective pressure for the development of antibiotic resistance in bacteria [65], which has a negative impact on public health [21,66]. We found all the APEC isolates to be resistant to ampicillin and tetracycline, and a high prevalence of resistance to chloramphenicol, erythromycin, enrofloxacin, norfloxacin, and ciprofloxacin. A 100% resistance of APEC isolates to ampicillin and tetracycline was also reported by Awad et al. [67]. In addition, Subedi et al. [17] and Ozawa et al. [68] reported higher percentages of APEC resistance to ampicillin, tetracycline, ciprofloxacin, and norfloxacin. For a long time, tetracycline has been used as a growth enhancer and a therapeutic agent in livestock production [69]; hence, the high level of resistance observed in this study is not surprising. In Bangladesh, tetracycline, and ampicillin have been used extensively to treat diseases in animals and humans (personal communication). However, the most alarming finding from our study is that all the APEC isolates were MDR in class. Similar findings were recorded in *E. coli* in poultry in Bangladesh [30,70,71,72], though the *E. coli* isolates had not been characterized as APEC. Subedi et al. [17] recorded 94% of APEC isolates as MDR in Nepal, similar to our findings. In South Korea, 94.1% of isolates were MDR in 2000–2005 [73], and 80.3% of APEC isolates were shown to be MDR in China [74]. Furthermore, several significant correlations among three fluoroquinolone class of antibiotics were identified in our current study. This finding is important, as researchers have strongly indicated that pairwise correlation can be linked with the development of resistance against other used antimicrobials [40].

Colistin is a last-resort antibiotic drug and should not be used for animal production. In our study, 11.1% of the isolates were resistant to colistin. Poultry are recognized as one of the major reservoirs and transmitters of colistin resistance [75]. Sobur et al. [32] reported colistin-resistant *E. coli* in poultry in Bangladesh, but these findings are much more frequent in China [76]. The indiscriminate use of antimicrobials is linked to a high prevalence of MDR strains in *E. coli* and in other enterobacteria [77]. The MDR and colistin-resistant APEC detected from layer birds and their surrounding air in the current study warn of serious health hazards for working personnel in farms and call for a further health study.

## 5. Conclusions

This is the first study in Bangladesh describing the detection of APEC in layers using molecular methods. In addition to internal organs, fecal samples and air samples were also found to carry APEC. Interestingly, all the APEC isolates were MDR in nature. Our current findings demonstrate that layer birds are potential reservoirs of antibiotic-resistant APEC, posing high public health risks to people who are exposed to them directly or indirectly. Based on importance, further studies should be employed to comprehend the dynamics and genetic diversity of antibiotic-resistant APEC associated with poultry, poultry farms, and their surroundings. However, considering the zoonotic significance, we propose the routine screening of APEC, targeting their virulence genes, for the early detection of avian colibacillosis and, hence, the protection of human health. Furthermore, the prevention of antimicrobial misuse, application of effective biosecurity measures, and adaptation of the one health approach are exigent measures for reducing AMR-related hazards.

## Figures and Tables

**Table 1:** Primers used in the detection of avian pathogenic *Escherichia coli* (APEC)-associated virulence genes.

Target Genes	Primer Sequence (5′–3′)	Amplicon Size (bp)	Annealing Temperature (°C)	References
16S rRNA	F: GACCTCGGTTTAGTTCACAGA R: CACACGCTGACGCTGACCA	585	55	[35]
*fimC*	F: GGGTAGAAAATGCCGATGGTG R: CGTCATTTTGGGGGTAAGTGC	496	59	[12]
*iucD*	F: ACAAAAAGTTCTATCGCTTCC R: CCTGATCCAGCTGATGCTC	692	55	
*papC*	F: TGATATCACGCAGTCAGTAGC R: CCGGCCATATTCACATAA	483	59	

**Table 2:** Prevalence of *E. coli* in layer farms.

Sample Source/Nature of Sample		No. of Samples Analyzed	No. Overall Analyzed	No. *E. coli* Positive Samples	Overall Positive for *E. coli*	Prevalence (%)	*p*-Value
Air		31	31	21	21	67.74	0.003
Feces		32	32	32	32	100	
Internal organs	Trachea	5	36	3	29	80.56	
	Intestine	8		7			
	Liver	14		11			
	Lung	7		6			
	Egg Yolk	2		2			
Total		99	99	82	82	82.83	

**Table 3:** Virulence genes in the isolated APEC.

Samples		Name of Positive Isolate	APEC-Associated Virulence Genes			No. of Positive Isolates (%)	*p*-Value
			*fimC*	*iucD*	*papC*		
Air (*n* = 31)		A1	+	+	+	5 (16.13)	˂0.001
		A2	+	-	+		
		A3	+	+	+		
		A4	+	+	+		
		A5	+	+	+		
Feces (*n* = 32)		F2	+	+	-	7 (21.87)	
		F3	+	+	+		
		F4	+	-	+		
		F5	+	-	+		
		F6	+	+	+		
		F7	+	-	-		
		F9	-	-	+		
Internal organs (*n* = 36)	Trachea (*n* = 5)	Io-T2	+	+	-	24 (66.67)	
		Io-T3	+	+	+		
	Intestine (*n* = 8)	Io-I2	+	+	-		
		Io-I3	+	+	-		
		Io-I4	+	-	-		
		Io-I5	+	+	-		
		Io-I6	+	+	-		
		Io-I7	+	-	+		
		Io-I8	+	-	-		
	Liver (*n* = 14)	Io-L1	+	+	-		
		Io-L2	+	+	-		
		Io-L3	+	-	-		
		Io-L4	+	-	-		
		Io-L6	+	-	-		
		Io-L7	+	-	-		
		Io-L9	+	+	-		
		Io-L10	+	+	-		
		Io-L13	+	+	-		
	Lung (*n* = 7)	Io-Lu1	+	-	-		
		Io-Lu2	+	-	-		
		Io-Lu5	+	-	-		
		Io-Lu7	+	+	-		
	Egg Yolk (*n* = 2)	Io-Y1	+	+	-		
		Io-Y2	+	+	-		
Total (*n* = 99)		36	35 (97.22%)	21 (58.33%)	12 (33.33%)	36 (36.36%)	
*p*-value			0.003				

A, Air; F, Feces; Io, Internal organs; T, Trachea; I, Intestine; L, Liver; Lu, Lung; Y, Egg yolk.

**Table 4:** Overall results of antibiotic sensitivity tests of APEC isolates.

SL No.	Isolate Name	CL	CIP	EX	NX	AMP	TE	S	GEN	C	E
1	A1	S	S	S	S	R	R	S	S	R	R
2	A2	S	S	S	S	R	R	I	I	R	R
3	A3	S	R	R	R	R	R	R	S	R	R
4	A4	S	R	R	R	R	R	R	S	R	R
5	A5	S	S	I	S	R	R	S	S	R	R
6	F2	S	R	R	R	R	R	S	R	R	R
7	F3	S	R	R	R	R	R	R	S	R	R
8	F4	S	R	R	R	R	R	I	S	R	R
9	F5	S	S	I	S	R	R	R	S	R	R
10	F6	S	S	I	S	R	R	R	S	R	R
11	F7	S	S	S	S	R	R	S	S	R	R
12	F9	S	S	S	R	R	R	S	S	R	R
13	Io-T2	S	S	S	S	R	R	S	I	R	R
14	Io-T3	S	R	R	R	R	R	R	S	R	R
15	Io-I2	S	R	R	R	R	R	S	S	R	R
16	Io-I3	S	R	R	R	R	R	S	S	R	R
17	Io-I4	S	I	R	I	R	R	S	S	R	R
18	Io-I5	R	R	R	I	R	R	S	R	R	R
19	Io-I6	S	S	R	S	R	R	S	S	R	R
20	Io-I7	S	I	R	R	R	R	S	R	R	R
21	Io-I8	S	R	S	S	R	R	S	S	R	R
22	Io-L1	R	R	R	R	R	R	S	S	R	R
23	Io-L2	S	R	R	R	R	R	S	S	R	R
24	Io-L3	S	R	R	R	R	R	I	S	R	I
25	Io-L4	S	S	S	S	R	R	I	S	R	R
26	Io-L6	S	S	S	S	R	R	S	S	R	R
27	Io-L7	S	S	S	S	R	R	S	S	R	R
28	Io-L9	S	S	S	S	R	R	S	S	R	R
29	Io-L10	S	R	R	R	R	R	S	S	I	R
30	Io-L13	S	R	R	R	R	R	S	S	R	R
31	Io-Lu1	S	R	R	R	R	R	I	S	R	R
32	Io-Lu2	R	R	R	R	R	R	R	S	R	R
33	Io-Lu5	S	S	S	S	R	R	S	S	R	R
34	Io-Lu7	S	I	I	S	R	R	S	S	R	R
35	Io-Y1	S	R	R	R	R	R	S	S	R	R
36	Io-Y2	R	S	I	S	R	R	S	S	R	R

A, Air; F, Feces; Io, Internal organs; T, Trachea; I, Intestine; L, Liver; Lu, Lung; Y, Egg yolk; CL, Colistin; CIP, Ciprofloxacin; EX, Enrofloxacin; NX, Norfloxacin; AMP, Ampicillin; TE, Tetracycline; S, Streptomycin; GEN, Gentamicin; C, Chloramphenicol; E, Erythromycin.

**Table 5:** Multidrug resistance profile of the isolated APEC.

Pattern No.	Antibiotic Resistance Pattern	No. of Antibiotics (Classes)	Isolate No.	No. of Isolates (%)
1	AMP, TE, C, E	4 (4)	A2, A5, F6, F7, Io-T2, Io-L4, Io-L6, Io-L7, Io-L9, Io-Lu5, Io-Lu7	11 (30.55)
2	AMP, TE, S, C, E	5 (5)	F5, F6	2 (5.55)
3	CL, AMP, TE, C, E	5 (5)	Io-Y2	1 (2.78)
4	EX, AMP, TE, C, E	5 (5)	Io-I4, Io-I6	2 (5.55)
5	NX, AMP, TE, C, E	5 (5)	F9	1 (2.78)
6	CIP, AMP, TE, C, E	5 (5)	Io-I8	1 (2.78)
7	CIP, EX, NX, AMP, TE, E	6 (4)	Io-L10	1 (2.78)
8	CIP, EX, NX, AMP, TE, C	6 (4)	Io-L3	1 (2.78)
9	CIP, EX, NX, AMP, TE, C, E	7 (5)	F4, Io-L2, Io-Y1, Io-I2, Io-I3, Io-L13	6 (16.67)
10	EX, NX, AMP, TE, GEN, C, E	7 (6)	Io-I7	1 (2.78)
11	CIP, EX, NX, AMP, TE, C, E	7 (5)	Io-Lu1	1 (2.78)
12	CIP, EX, NX, AMP, TE, S, C, E	8 (6)	A3, A4, F3, Io-T3	4 (11.11)
13	CL, CIP, EX, AMP, TE, GEN, C, E	8 (7)	Io-I5	1 (2.78)
14	CIP, EX, NX, AMP, TE, GEN, C, E	8 (6)	F2	1 (2.78)
15	CL, CIP, EX, NX, AMP, TE, C, E	8 (6)	Io-L1	1 (2.78)
16	CL, CIP, EX, NX, AMP, TE, S, C, E	9 (7)	Io-Lu2	1 (2.78)
	Total			36

CL, Colistin; CIP, Ciprofloxacin; EX, Enrofloxacin; NX, Norfloxacin; AMP, Ampicillin; TE, Tetracycline; S, Streptomycin; GEN, Gentamicin; C, Chloramphenicol; E, Erythromycin; A, Air; F, Feces; Io, Internal organs; T, Trachea; I, Intestine; L, Liver; Lu, Lung; Y, Egg yolk.

**Table 6:** Pairwise correlations between resistance to antimicrobials of APEC isolated from layer chickens (*n* = 36) ^AB^.

	CL	CIP	EX	NX	AMP	TE	S	C	GEN	E
CL	1.0000	-	-	-	-	-	-	-	-	-
CIP	0.1195	1.0000	-	-	-	-	-	-	-	-
EX	0.2345	0.6625 *	1.0000	-	-	-	-	-	-	-
NX	0.1383	0.8315 *	0.6203 *	1.0000	-	-	-	-	-	-
AMP	-	-	-	-	-	-	-	-	-	-
TE	-	-	-	-	-	-	-	-	-	-
S	−0.0625	0.1195	0.2132	0.1581	-	-	1.0000	-	-	-
C	-	-	-	-	-	-	-	-	-	-
GEN	0.1136	0.0136	−0.0823	0.0359	-	-	−0.1136	-	1.0000	-
E	-	-	-	-	-	-	-	-	-	-

Here, (*) indicates a significant correlation with a *p* value less than or equal to 0.00625 (*p* ≤ 0.00625). ^A^ CL, Colistin; CIP, Ciprofloxacin; EX, Enrofloxacin; NX, Norfloxacin; AMP, Ampicillin; TE, Tetracycline; S, Streptomycin; C, Chloramphenicol; GEN, Gentamicin; E, Erythromycin. ^B^ Spearman rank correlation, with a Bonferroni correction (*α*/8) to adjust for multiple comparisons, was performed to determine the pairwise correlation between the resistance to antibiotics.
